# Biochemical and computational study of an alginate lyase produced by *Pseudomonas aeruginosa* strain S21

**DOI:** 10.22038/ijbms.2020.37277.8874

**Published:** 2020-04

**Authors:** Firoozeh Piroozmand, Parinaz Ghadam, Mahboobe Zarrabi, Ahya Abdi-Ali

**Affiliations:** 1 Department of Biotechnology, Faculty of Biological Sciences, Alzahra University, Tehran, Iran; 2 Department of Microbiology, Faculty of Biological Sciences, Alzahra University, Tehran, Iran

**Keywords:** Alginate, Alginate lyase, Computational biology, Enzyme assay, Pseudomonas aeruginosa, Strain S21

## Abstract

**Objective(s):**

Alginates play a key role in mucoid *Pseudomonas aeruginosa* colonization, biofilm formation, and driving out of cationic antibiotics. *P. aeruginosa* alginate lyase (AlgL) is a periplasmic enzyme that is necessary for alginate synthesis and secretion. It also has a role in depolymerization of alginates. Using AlgLs in cystic fibrosis patients along with antibiotics enhances bacterial killing and host healing. In this study, we investigated the different biochemical properties of a newly isolated AlgL from *P. aeruginosa* S21 to complete the databank of AlgLs

**Materials and Methods:**

The enzyme was extracted from the periplasmic space of the bacteria by the heat shock method. Using the TBA method, the enzyme activity and biochemical properties were assessed. The mutability of *P. aeruginosa* S21 AlgL to increase its thermal stability was investigated. The most favorable mutations were studied computationally. The molecular dynamics simulation (MDS) package GROMACS was used for determining the effect of S34R mutation on enzyme’s thermal stability.

**Results:**

Data showed that this enzyme has the best activity at 37 ^°^C and pH 7.5 and it can degrade mannuronate blocks, guluronate blocks, and sodium alginate. After 7 hr at 80 ^°^C, 45% of the enzyme activity was retained. This enzyme needed 15 min to completely degrade accessible sodium alginate. Tris buffer, pH 8.5 and Britton-Robinson buffer, pH 7.0 were the preferable buffers for the enzyme activity. MDS of native and mutated enzymes showed desirable results.

**Conclusion:**

*P. aeruginosa* S21 AlgL can be used in medical and industrial applications to degrade alginates.

## Introduction

Bacterial alginate lyases (AlgLs), consisting of several polysaccharide lyases, have important industrial, medical, agricultural, and biotechnological applications (1). This enzyme contributes to the synthesis and degradation of alginates. AlgLs can be found in organisms that have alginate metabolizing systems or biosynthetic systems (2). *Pseudomonas aeruginosa* is a Gram-negative bacterium that grows in soil, marshes, coastal marine habitats, and also plant and animal tissues. *P. aeruginosa* alginate is a linear polymer composed of D-mannuronate and L-guluronate with β (1→4) linkages that has O-acetyl groups on 2 and/or 3 positions of mannuronic acid residues (3, 4). Acetylation of the alginate polymer changes its water solubility and binding to different ions. Mucoid clinical strain forms microcolonies on steel surfaces whereas the mutant with acetylation defect cannot form cellular groups. Alginate enzymatic degradation by AlgL prevents the formation of the mucoid parent strain (5). Alginates exist in three forms: homopolymeric G-blocks or polyguluronate [poly(G)], homopolymeric M-blocks or polymannuronate [poly(M)], and heteropolymeric G/M blocks (6).

In *P. aeruginosa*, alginate is produced in the form of an exopolysaccharide capsule that is attached weakly to the bacterial cells, so most of it is found in the culture supernatant (7).

The role of alginates as barriers to antibiotic penetration has been verified by researchers (8). AlgL is an endolytic enzyme, and its main products are disaccharides and trisaccharides. AlgLs depolymerize alginates by breaking the 1→4 glycosidic bond via β elimination and produce a variety of oligosaccharides with unsaturated uronic acid at the non-reducing end and unsaturated uronic acid monomers (3, 9).

AlgLs have a preference for L-guluronic acid or D-mannuronic acid residues. According to the substrate specificities, AlgLs are grouped into three types: PolyM-specific lyases (EC 4.2.2.3), PolyG-specific lyases (EC 4.2.2.11), and bifunctional lyases that can degrade both PolyM and PolyG. Until now, most of the AlgLs have been determined to be polyM-specific lyases. Even though they can be categorized as polyM-, polyG-, and polyMG-specific lyases, they usually show low activity against the other blocks (10).

In the present work, we investigated the different biochemical characteristics of *P. aeruginosa* S21 AlgL and tried to find a relation between its protein sequence and the observed biochemical features. We also aimed to discover the residues of the enzyme active site responsible for the interaction with the alginate molecule. A few mutations were suggested to raise the thermal stability of AlgL.

## Materials and Methods

***Molecular studies***

The mucoid *P. aeruginosa* strain S21, which had been isolated from burn patients, was kindly provided by Dr. A. Abdi-Ali, maintained in skim milk at -70 ^°^C, and grown on an LB medium overnight at 37 ^°^C. A few grown colonies were transferred to YTG medium (0.5% yeast extract, 1% tryptone, and 0.2% glucose) and cultured in a shaker incubator at 37 ^°^C, 180 rpm for 10 hr. The biomass was collected by centrifugation at 4 ^°^C, 6500 g for 25 min (11). 

16S rRNA gene PCR was performed using primers: 27F ((5’-AGAGTTTGATCCTGGCTCAG-3’) and 1492R (5’-GGCTACCTTGTTACGACTT-3’); the respective band appeared on the agarose gel. The *algL* gene was also amplified by PCR using Primers that had been designed and used by Tavafi in 2017 based on *P. aeruginosa* PAO1: FC (5’-ATATGAATTCATGAAAACGTCCCACCTGAT-3’) and RC (5’ -ATATAAGCTTTCAACTTCCCCCTTCGC-3’). The PCR product was extracted from the agarose gel using a GF-1 GEL DNA Recovery Kit and sequenced by Bioneer Corporation (Daejeon, South Korea). The sequence was blasted at NCBI and submitted to GeneBank (accession number MF461642) (12).

***Biochemical studies***

The Heat shock method was used to extract the enzyme from the periplasmic space of the bacteria. The pellet was suspended in 0.05 M Tris-HCl buffer containing 0.2 M MgCl_2_ (pH 7.5) and kept at 37 ^°^C for 10 min. Then, it was immediately transferred to 0 ^°^C and remained at that temperature for 15 min. This procedure was repeated four times. Afterward, the suspension was centrifuged at 4 ^°^C and 6500 g for 15 min and the supernatant was collected as a crude enzyme extract (13).

AlgL activity was measured by the thiobarbituric acid (TBA) method. The enzyme extract, assay buffer (Tris-HCl 0.03 M, NaCl 0.5 M, and MgCl_2_ 0.009 M) pH 8.5, and sodium alginate were mixed together with a ratio of 2:2:1 in a 100 µl volume. The 125 µl periodic acid solution in 0.125 N H_2_SO_4_ was added to the mixture and placed at 37 ^°^C for 20 min. Subsequently, 250 µl of the 2% sodium arsenite solution in 0.5 M HCl was added to the mixture, and after 2-min incubation at room temperature, 1 ml of the 0.3% thiobarbituric acid solution in the assay buffer pH 2.0 was added with gentle shaking and the final mixture was placed in boiling water for 10 min. Eventually, immediately after the solution became chilled, its absorbance was measured at 548 nm by a spectrophotometer and reported as the enzyme unit. One unit of the enzyme activity is considered as the amount of the enzyme needed for producing 1 nmol of β-formylpyruvic acid per minute in one ml at 37 ^°^C. 0.29 absorbance at 548 nm is considered as 10 units of AlgL (14-17). 

To determine the optimum operating time of the enzyme, the routine TBA method was used, but the crude enzyme extract, the assay buffer and the sodium alginate were mixed and put at 37 ^°^C (which is the preferred temperature for most of the AlgLs of this species) for 0, 15, 30, 45, 60, and 75 min. Then, the 0.025 M pyruvic acid solution in 0.125 N H_2_SO_4_ was added to the samples and later steps were performed as before. The experiment was accomplished with three repeats (16, 18). 

To study the optimum temperature for AlgL activity, the cell extract containing the enzyme was put on ice, and then equal amounts of it were taken and kept for one hour at 10, 37, 60, and 80 ^°^C. The enzyme activity before any temperature treatment was considered as 100%. After one hour at those temperatures, all samples were put on ice. Then the TBA method was used for assessing all samples (19, 20). 

Since the enzyme showed suitable stability at 80 ^°^C as an extreme temperature, the cell extract containing AlgL was incubated at 80 ^°^C and after 1, 2, 3, 4, 5, 6, and 7 hr at that temperature, each time some of the sample was taken and placed on ice and assayed by the TBA method for three repeats (20, 21). 

For this investigation, the sodium alginate and its derivatives (M- and G- blocks) that had been provided by Tavafi based on the Haug *et al.* method were used as substrate (22). Enzyme activity assay procedure was accomplished for each substrate for three repeats by the TBA method (23, 24). 

To investigate the optimum pH for the enzyme activity assay, the Britton-Robinson buffer was used instead of the assay buffer used in the routine TBA method. Buffer was prepared with boric acid, acetic acid, and phosphoric acid, all with the final concentration of 0.04 M in an aqueous solution. The pH range, including 2.5, 4, 5.5, 7, 8.5, 10, and 11.5, was prepared. AlgL activity was measured in all these pHs by the TBA method (25). 

To study AlgL stability against different pHs, the cell extract in distilled water was mixed with Britton-Robinson buffer with pHs 2, 4, 7, and 11. At 1, 2.5, 3, 5.5, and 6 hr after remaining in those pHs, enzyme activity was assayed by the TBA method (21, 24). All other conditions were at their optimum states determined before.

***Computational studies***

To investigate the interaction between AlgL and the alginate molecule, their three dimensional (3D) structures were needed. The alginic acid disaccharide code 39944744 was selected from the ZINC database (zinc.docking.org) as a ligand. Since no 3D structures for the three enzymes were found on PDB (Protein Data Bank), therefore their 3D structures were modeled using the Modeller 9.17 software. One hundred models were built for each enzyme. The most suitable models with the least value of MODELLER objective function were opted for further investigation (26). The accuracy of the built models was studied using the Qmean web server (www.swissmodel.expasy.org/qmean/)(27). The model refinement was performed by the 3Drefine web server (sysbio.rnet.missouri.edu/3Drefine/) and the best refined models with this webserver were refined again by another web server, Galaxyrefine (galaxy.seoklab.org/cgi-bin/submit.cgi?type=REFINE) (28, 29).

***Mutation studies***

 In order to find the enzymes’ crucial residues contributing to interaction with the substrate, the AutoDock 1.5.6 software package was used for docking simulation. ConTEXT and UCSF Chimera 1.12 software packages were also employed for the protein and ligand preparation before docking. One hundred ligand conformers were built for each enzyme. The conformers located in the active site of the enzymes, which had been defined with the CASTp server, with the least binding energy and the most hydrogen bonds formed between the enzyme and the ligand, were selected as correct conformers. Complexes related to these conformers were built as PDB files. Residues involved in the alginic acid degradation with hydrogen bonds or hydrophobic bonds in the enzyme active site were displayed using Ligplot. Since these residues do the main activity of the enzyme, they should remain intact during mutation. 

AlgL contains a signal peptide that is processed before being located in the periplasmic space. The signal peptide of the enzyme was predicted using SignalP 4.1 Signalblast, Phobius, Signal-3L 2.0. Disulfide bonds in enzymes are an important index of their resistance at high temperatures. Every cysteine and the probable disulfide bonds were analyzed using Cyscon, Dlpro, DISULFIDE and DiANNA. The secondary structure of the enzyme was studied using RaptorX, SPOT, CSpritz, and PSIPRED based on its protein sequence.

The residues with high mutability in the enzyme structure were investigated using HotSpot Wizard 2.0. Actually, the residues that had high mutability, not located in the enzyme active site and not necessary for the enzyme activity, were chosen to be mutated. The residues located on the protein surface were preferred because if any mutation occurs, the new residue will have no clashes with adjacent residues. According to several studies, the mutations in the residues with high mutability and without any disadvantages were suggested for the enzyme activity optimization. The conserved regions among AlgLs provided by articles were searched in the protein sequence. These conserved regions are mostly related to the different substrate specificities of AlgLs. As these regions are conserved during generations and they give the enzymes their special features, none of them should undergo mutation. 

A few mutations were suggested based on all of the results attained. The effect of suggested mutations on the enzyme stability was analyzed using SDM, MCSM, and DUET. Desirable mutations that could increase the enzyme stability were chosen. Selective residues were mutated using UCSF Chimera 1.12. The mutated enzymes were docked with the previous substrate and the complexes were attained. 

***Molecular dynamics simulation***

Finally, a molecular dynamics simulation (MDS) was performed to assess the enzyme-substrate complex structure, the intermolecular interactions, and comparing mutant complex with wild-type. Wild-type *P. aeruginosa* S21 AlgLs in complex with alginic acid disaccharide and mutant strain of this bacteria with the mutation S34R in complex with alginic acid disaccharide were simulated using GROMACS 5.1.4 at 37 ^°^C (T=310 K) and 80 ^°^C (T=353 K) for 5 ns. GROMOS96 43A1 force field was used for residues of the enzyme and GROMOS87 for HETATOM molecules provided by the PRODRG server.

## Results

***Finding the optimum time for enzyme-substrate reaction***

**Figure 1.**
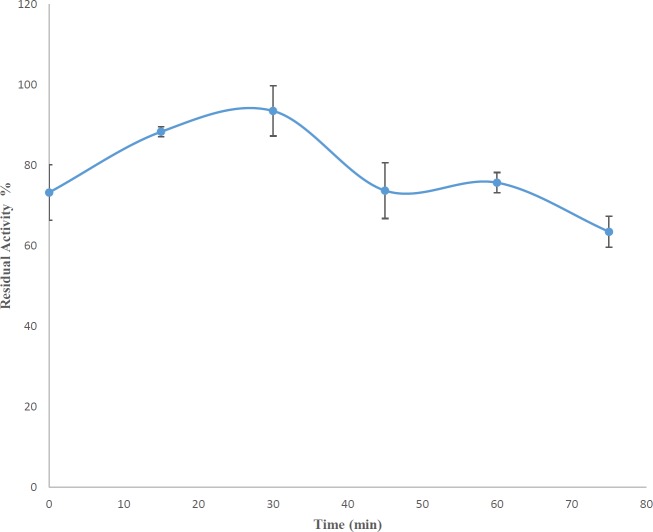
*Pseudomonas aeruginosa* S21 AlgL residual activities at different incubation times. One-way ANOVA with *post hoc* Tukey’s test showed that the value of residual activities at 15 and 30 min after incubation does not make sense (*P-value*>0.05); so, we chose 15-min incubation time for the rest of our study

The optimum time required for *P. aeruginosa* S21 AlgL to completely degrade sodium alginate was 15 min (Figure 1).

***Investigating the optimum temperature for AlgLs activity***

**Figure 2:**
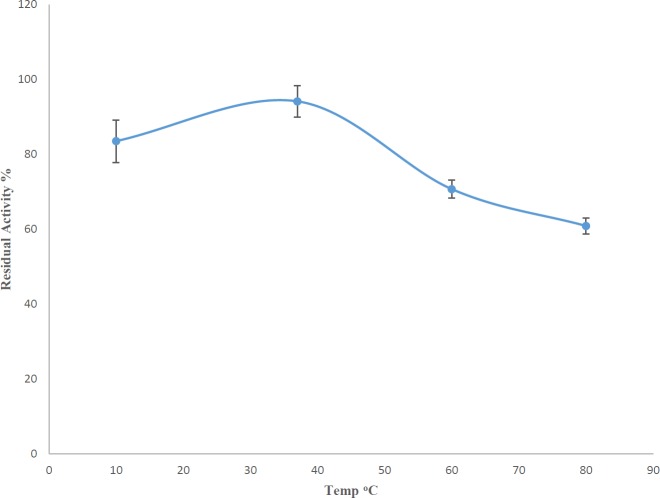
Residual activities of *Pseudomonas aeruginosa* S21 AlgL after remaining for 1 hr at different temperatures

The study of the temperature preference of *P. aeruginosa* S21 AlgL showed that there was a 20% loss in the enzyme activity after one hour at 10 ^°^C. At 37 ^°^C, it could retain the maximum activity compared with other temperatures (one-way ANOVA with *post hoc* Tukey’s test, *P*-value<0.05) (Figure 2). This result had not been predicted. Hence, in this study, a decision was made not to keep the enzyme in the refrigerator but a -20 ^°^C deep freezer.

***Study of AlgLs stability at 80*** ^°^ ***C***

**Figure 3:**
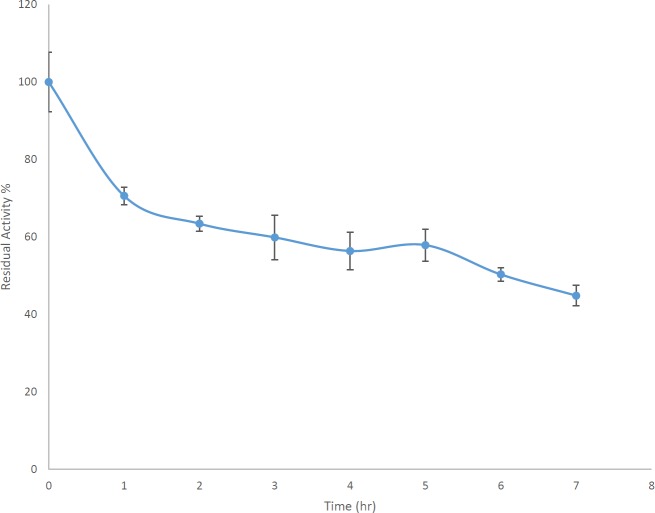
*Pseudomonas aeruginosa* S21 AlgL resistance during 7 hr at 80 ^°^C

Since it had been illustrated that *P. aeruginosa* S21 AlgL could retain approximately 70% of its activity at 80 ^°^C, the enzyme stability was assessed for 7 hr at this temperature. After 7 hr, 45% of the enzyme activity was preserved (Figure 3).

***Studying the AlgLs substrate specificity***

**Figure 4:**
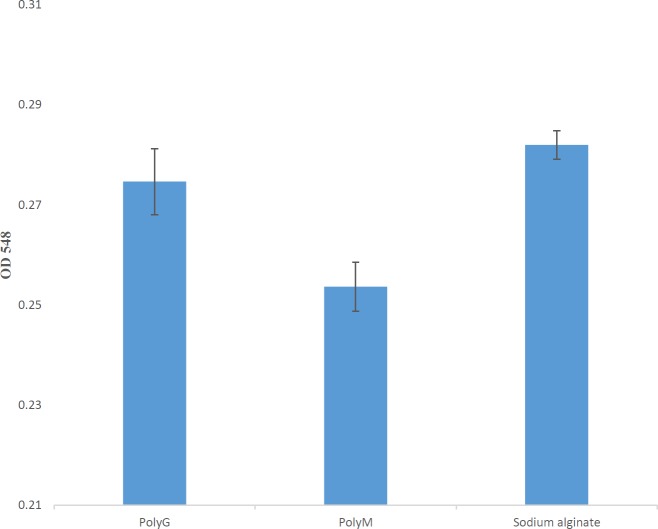
*Pseudomonas aeruginosa* S21 AlgL activity on three kinds of substrates

*P. aeruginosa* S21 AlgL had effects on three different substrates: sodium alginate, M-, and G- blocks, more on polyMG sodium alginate, and on G-blocks more than M-blocks (one-way ANOVA with *post hoc* Tukey’s test, *P*-value<0.05) (Figure 4). Most of the *P. aeruginosa* AlgLs show more activity on M-blocks than G-blocks or large differences exist in their preferences for a particular block.

***Investigating the pH effect on enzyme activity***

**Figure 5:**
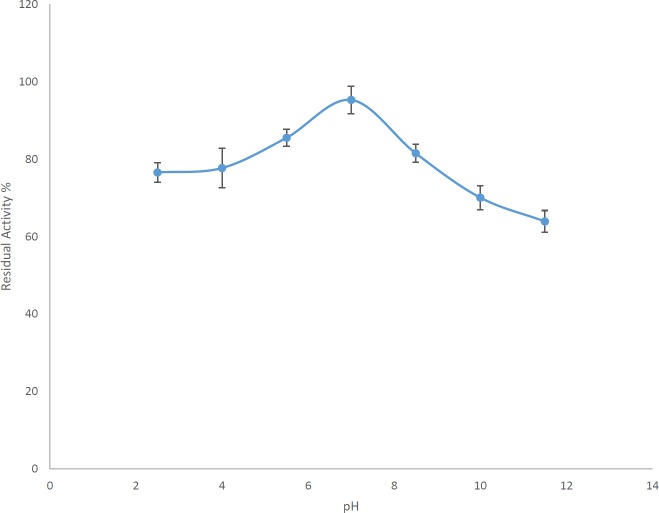
Effects of the assay buffer with different pHs on the *Pseudomonas aeruginosa* S21 AlgL assay by the TBA method

After using the Britton-Robinson buffer with different pHs, instead of the routine assay buffer, the highest activity was observed at pH 7.0. It should be noted that this optimum pH is for the Britton-Robinson buffer. In the routine assay of the enzyme activity by the TBA method, the enzyme had more activity with the assay buffer at pH 8.5 than this buffer at pH 7.0 (one-way ANOVA with *post hoc* Tukey’s test, *P*-value<0.05). So, the buffer type is another factor affecting the AlgL activity in the TBA method (Figure 5).

***Studying enzyme stability against different pHs***

**Figure 6:**
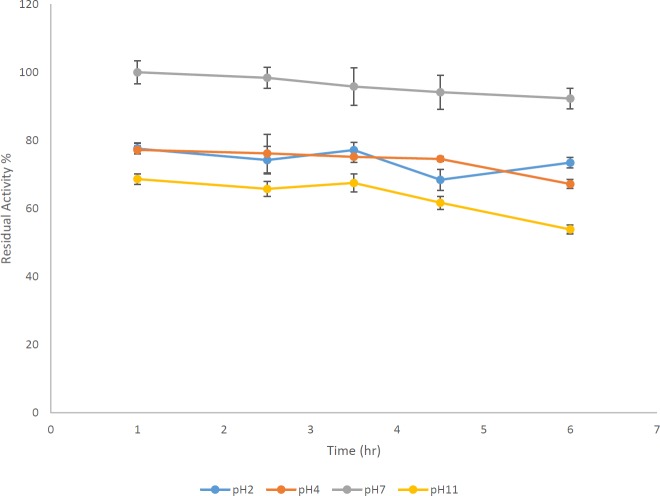
Resistance of *Pseudomonas aeruginosa* S21 AlgL during 6 hr at four different pHs

*P. aeruginosa* S21 AlgL had retained 73%, 67%, 92%, and 53% of its activity after remaining for 1 hr at pHs 2, 4, 7, and 11, respectively (one-way ANOVA with *post hoc* Tukey’s test, *P*-value<0.05) (Figure 6).

***Mutation studies***

 After searching for the signal peptide, it was found that *P. aeruginosa* S21 AlgL has an excision site between the residues 16 and 17. Since this region would be separated from the final enzyme, no mutations were suggested for it. The docking results demonstrated that Lys 51, Lys126, Lys 233, Lys 130, Asn 243, and Thr 299 participated in hydrogen interactions with alginate molecule in *P. aeruginosa* S21 AlgL active site. There were also residues in the enzyme’s active site with a role in hydrophobic interactions with alginate molecules Asn 185, Asn 186, Ile 184, Tyr 189, and Leu 239. These residues were not considered for mutation since they carry out the main activity of the enzymes. Searching for the conserved regions among AlgLs such as NNHSYW, WLEPXCXLY, RXELR, YFKAGXYXQ, YXRSELREM, YXRESLREM, RSEL, and Q (I/V) H indicated that the AlgL studied in this research has NNHSYW in the center of its protein sequence, which is related to PolyM lyase activity as observed in the lab. It also contained WLEPYCALY at its C-terminus, which is, again, related to PolyM lyase activity (30).

The best-suggested mutations were tested using the SDM server to check if they were stabilizing or destabilizing. Mutations D97R, S197A, S72R, and S34R for *P. aeruginosa* S21 AlgL were predicted as stabilizing.

***Molecular dynamics simulation***

**Figure 7:**
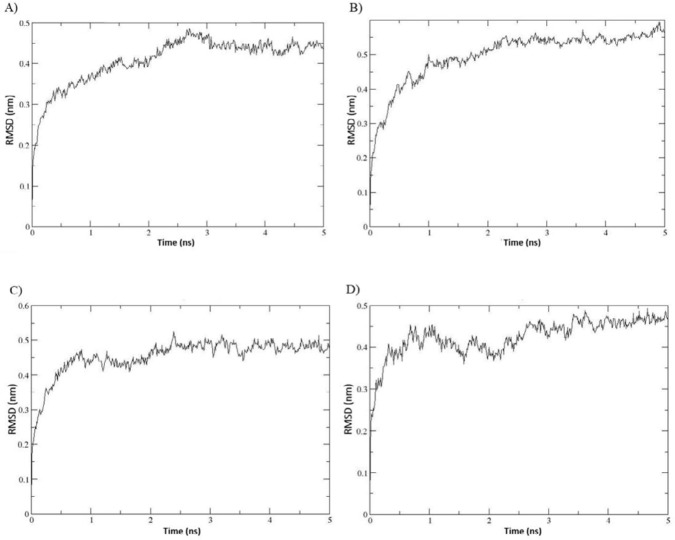
The RMSD plots of the wild-type *Pseudomonas aeruginosa* S21 AlgL in complex with alginic acid disaccharide at 37 ^°^C during 5 ns MDS(A) and mutated AlgL complex with S34R mutation (B). The wild-type complex at 80 ^°^C (C) complex with S34R mutation at 80 ^°^C (D)

**Figure 8:**
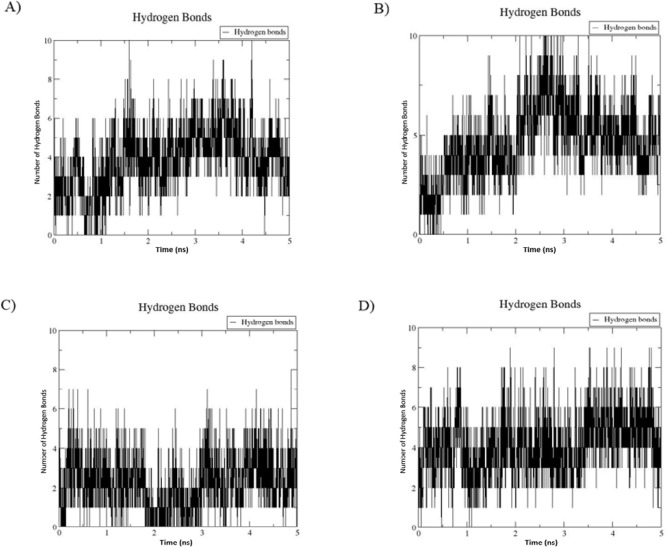
The hydrogen-bond plots formed between the *Pseudomonas aeruginosa* S21 AlgL and the substrate during MDS at 37 ^°^C for 5 ns (A), S34R mutated at 37 ^°^C (B). wild-type at 80 ^°^C (C) and S34R mutated at 80 ^°^C (D)

MDS results for the S34R mutation in *P. aeruginosa* S21 AlgL in complex with alginate molecule were desirable. The root mean square deviation (RMSD) values of the backbone atoms of the wild-type and mutant enzymes complex with the alginate molecule were monitored during MDS at 37 ^°^C and 80 ^°^C, indicating the 3D structural conformations stability. The RMSD profiles of both complexes reached equilibrium after approximately 3.5 ns MDS and remained stable until the end of the simulation, which represents that the system was equilibrated (Figure 7). Hydrogen bonding plots of the wild-type and the S34R mutant alginate lyases in complex with the alginate molecule were also examined. A minimum of one and a maximum of 10 H-bonds were maintained between the *P. aeruginosa* S21 alginate lyase and the ligand during simulation at 37 ^°^C, and a minimum of zero and maximum of 6 H-bonds at 80 °C. In the S34R mutant alginate lyase complex with the ligand, the minimum of one and the maximum of 10 H-bonds were preserved at 37 ^°^C, and a minimum of one and a maximum of 8 H-bonds at 80 ^°^C. So, hydrogen-bonding interactions between the mutant enzyme and the substrate molecule were preserved more at 80 ^°^C compared with that in the wild-type (Figure 8).

## Discussion

Despite all their similarities, AlgLs have many differences in their biochemical properties such as optimum temperature, optimum pH, substrate specificity, molecular weight, structure, and stability at different temperatures and pHs. These differences are present not only among different species but also different strains. AlgLs extracted from various strains of *P. aeruginosa* have been investigated in earlier studies (31, 32). Thus, to complete the databank of AlgLs, another strain of *P. aeruginosa* was chosen, and by extracting its AlgL, its properties were studied. AlgLs produced by the different strains of *P. aeruginosa* show different characteristics. For example, *P. aeruginosa* 293 AlgL, which was studied by Zali *et al.*, could retain all its activity even after remaining at 10 ^°^C for 10 days, but the AlgL reported in the present study had lower resistance at 10 ^°^C. Another difference between the two AlgLs was that the *P. aeruginosa* S21 enzyme needed 15 min to degrade sodium alginate completely, but the *P. aeruginosa* 293 AlgL could degrade all the substrate immediately after confronting it.

After remaining for 6 hr at 80 ^°^C, *P. aeruginosa* S21 AlgL retained about 50% of its activity, whereas* P. aeruginosa* 293 AlgL retained about 70% of its activity (33); 35% of *P. aeruginosa* TAG48 AlgL activity was retained after remaining for 4 hr at 80 ^°^C (31).

*P. aeruginosa* S21 had an effect on the three different alginate substrates: sodium alginate, polyM, and polyG; it demonstrated more effect on the polyG substrate than polyM. Since most of the *P. aeruginosa* AlgLs are M lyases, it can be said that this is an interesting trait of the *P. aeruginosa* S21 AlgL. *P. aeruginosa* TAG48 and *P. aeruginosa* 293 alginases have shown similar activity toward these substrates (31). The protein sequence of *P. aeruginosa* S21 AlgL demonstrated that AlgL has 354 residues. The molecular weight predicted for the enzyme was 39.44. The molecular weight that is about 40 kDa is also related to the specific degrading activity against polyM and NNHSYW conserved region (6).

A lot of research has been done to determine the role of the amino acid sequence in the thermal stability of a protein. There is a high demand for thermal resistant enzymes in industry and biotechnology because they are more stable and, hence, more suitable for extreme environments. Thermal resistant proteins can be produced by both thermophiles and mesophils. Although thermophilic microorganisms are a potential source of these proteins, most of them are derived from mesophils (34, 35). Mutations that increase the thermal stability of a protein enhance its thermodynamic resistance or unfolding ratio by reducing the difference between the folded and unfolded states.

At first, we decided to compare the protein sequence of *P. aeruginosa* S21 AlgL to naturally thermostable AlgLs that had been characterized before; very low similarity was obtained. Hence, it is not possible to use known thermal resistant alginases as a template to reach this goal. There are patterns for thermal stability. Arg has been found as the N-terminal amino acid in proteins at temperatures higher than 70 ^°^C. The major features of thermostable proteins include greater hydrophobicity, more compaction, deletion or shortage of loops, smaller and fewer cavities, less thermolabile residues, more helical content, more polar surface, more hydrogen bonds, and more salt bridges. The thermostability of the enzymes can be enhanced by single nucleotide substitutions (36). Arg and Tyr are notably more abundant and Cys and Ser less abundant in thermophilic proteins. In this research, we tried to substitute serine in high mutability regions with arginine. Computational studies indicated more stability of the mutated enzymes than the wild-type. (37, 38). One suggested mutation, S34R (at N-terminus), in *P. aeruginosa* S21 AlgL was the only mutation that was investigated by MDS and showed favorable results.

## Conclusion

The *P. aeruginosa* AlgL acted in a wide range of pHs and temperatures. According to the observed substrate specificity, *P. aeruginosa* S21 AlgL can be used on complex alginate substrates and degrade polyguluronate, polymannuronate, and G/M blocks. Consequently, it can be a suitable candidate in medical and industrial applications to degrade alginates. This research also showed that according to computational results, the substitution of thermolabile residues such as Ser on the surface of the enzyme with suitable residues might enhance its thermostability.
